# Effects of histamine H1 receptor signaling on glucocorticoid receptor activity. Role of canonical and non-canonical pathways

**DOI:** 10.1038/srep17476

**Published:** 2015-12-04

**Authors:** Carlos Daniel Zappia, Gina Granja-Galeano, Natalia Fernández, Carina Shayo, Carlos Davio, Carlos P. Fitzsimons, Federico Monczor

**Affiliations:** 1 Laboratorio de Farmacología de Receptores, Cátedra de Química Medicinal, Facultad de Farmacia y Bioquímica, Universidad de Buenos Aires. Buenos Aires, Argentina; 2 Laboratorio de Patología y Farmacología Molecular, Instituto de Biología y Medicina Experimental, CONICET, Buenos Aires, Argentina; 3 Neuroscience Programme, Swammerdam Institute for Life Sciences, University of Amsterdam, SciencePark 904, 1098XH Amsterdam, The Netherlands; 4 Instituto de Investigaciones Farmacológicas, ININFA. Universidad de Buenos Aires—Consejo Nacional de Investigaciones Científicas y Técnicas, CONICET. Buenos Aires, Argentina

## Abstract

Histamine H1 receptor (H1R) antagonists and glucocorticoid receptor (GR) agonists are used to treat inflammatory conditions such as allergic rhinitis, atopic dermatitis and asthma. Consistent with the high morbidity levels of such inflammatory conditions, these receptors are the targets of a vast number of approved drugs, and in many situations their ligands are co-administered. However, this drug association has no clear rationale and has arisen from clinical practice. We hypothesized that H1R signaling could affect GR-mediated activity, impacting on its transcriptional outcome. Indeed, our results show a dual regulation of GR activity by the H1R: a potentiation mediated by G-protein βγ subunits and a parallel inhibitory effect mediated by Gαq-PLC pathway. Activation of the H1R by its full agonists resulted in a composite potentiating effect. Intriguingly, inactivation of the Gαq-PLC pathway by H1R inverse agonists resulted also in a potentiation of GR activity. Moreover, histamine and clinically relevant antihistamines synergized with the GR agonist dexamethasone to induce gene transactivation and transrepression in a gene-specific manner. Our work provides a delineation of molecular mechanisms underlying the widespread clinical association of antihistamines and GR agonists, which may contribute to future dosage optimization and reduction of well-described side effects associated with glucocorticoid administration.

Inflammation-related diseases present a great challenge in current medicine due to, among other factors, their high morbidity. Consistently, the histamine H1 receptor (H1R) and the glucocorticoid receptor (GR) are targets with the most number of drugs approved^1^, and they’re often used in combination therapies^2^,^3^. Glucocorticoids (GC) are highly effective in combating inflammation in the context of a variety of diseases, such as asthma, allergic rhinitis (AR), atopic dermatitis (AD) and rheumatoid arthritis (RA)^4^. GC are involved in critical processes such as growth, reproduction, central nervous system and cardiovascular functions and immune and inflammatory actions as well as cell proliferation and survival. Their anti-inflammatory and immunomodulatory effects make these steroids the standard therapy to treat numerous autoimmune, inflammatory and allergic disorders, such as, asthma, rheumatoid arthritis and AR among others. Unfortunately, chronic exposure to these agents becomes a problem for therapy resulting in a wide set of undesirable effects^5^.

There are different possible approaches to improve GC’s beneficial/adverse effect ratio, going from chemical optimization to the development of selective glucocorticoid receptor agonists (SEGRAs) based on the assumption that ligands which only induce gene transrepression and not transactivation should have a better therapeutic profile. Alternatively, add-on therapies present another way to control adverse effects by reducing GC dose and combining it with a different drug with anti-inflammatory activity.

In most tissues, both the beneficial and the adverse effects of GC are dose-dependent and mediated by activation of the GR^6^. The GR is a ligand-activated transcription factor, which once activated by hormone binding, homodimerizes, translocates to the nucleus and binds to specific target sequences in the DNA, called GC-response elements (GREs), thereby modulating gene transcription^7^. However, transrepression and transactivation of specific genes induces beneficial and adverse effects, respectively. Traditionally, it has been accepted that GC’s anti-inflammatory activity may be due to GR interaction with transcription factors, e.g. NF-κB, and inhibition of gene expression (transrepression), while the activation of gene transcription by GR binding to GREs (transactivation) may be responsible of metabolic effects and adverse effects at pharmacological doses^6^,^8^,^9^. However, new insights into GC’s anti-inflamatory action have revealed that transactivation plays a central role in anti-inflamation, as well^10^,^11^. In this context, the reduction of GR-mediated transactivation by extracellular molecules can play an important role in the improvement of GC’s therapeutic profile.

The H1R is one of the four distinct G-protein coupled receptors that mediate histamine responses in the body. The binding of histamine to the H1R results in dissociation of the Gαq/11 subunit from the Gβγ dimer, resulting in the activation of several downstream effectors that lead to the modulation of membrane phosphoinositide metabolism and intracellular calcium levels. Since histamine pro-inflammatory effects are largely mediated by its action on H1R, antagonists of this receptor are often used to treat several inflammatory-related conditions. Improtantly, many of these clinically used antihistamines are not antagonists but inverse agonists, therefore decreasing H1 receptor constitutive activity^12^,^13^.

GR activity can be modulated intracellularly at several levels, including protein-protein interaction and post-translational modifications, such as, phosphorylation, ubiquitination, acetylation and sumoylation that affect ligand affinity, receptor localization, transcriptional activity and turnover^14^. Some previous studies have explored the possibility to modulate GR signaling through the activation or inhibition of GPCR-mediated signaling, demonstrating crossregulation between GR and the β2-adrenergic, somatostatin and melatonin GPCRs. Mechanistically, epinephrine and norepinephrine enhance GR activity via a Gβγ/PI3K/PKB pathway^15^, while somatostatin and melatonin suppress GR activity through Gβγ and Gαi proteins respectively^16^,^17^. Somehow surprisingly, due to the common therapeutic association with GR agonists, no studies have been conducted to characterize the effects of H1R-activated intracellular pathways on GR activity. Hence, the aim of this work was to study how H1R signaling induced by its agonists or inverse agonists modulates GR-mediated transcriptional activity induced by its agonists dexamethasone and corticosterone. Our results show a complex dual regulation of GR activity by the H1R, consisting of a potentiation of dexamethasone effects mediated by G-protein βγ subunits and Jun kinase-mediated GR phosphorylation and a parallel inhibition of dexamethasone effect, mediated by the canonical Gαq-PLC-Rac pathway. When the H1R is activated by its natural agonist, histamine, the simultaneous triggering of both pathways results in a composite activating effect. Conversely, when H1R is bound by inverse agonists, the canonical inhibitory Gαq-PLC-Rac pathway is repressed, resulting in a potentiation of GR-mediated transcriptional activity. The characterization of signaling mechanisms underlying these complex interactions, as well as the discussion of their possible clinical implications, are the main purpose of the present work.

## Results

### Effect of H1R activation on dexamethasone- and corticosterone-induced GR activity

**Figure 1:**
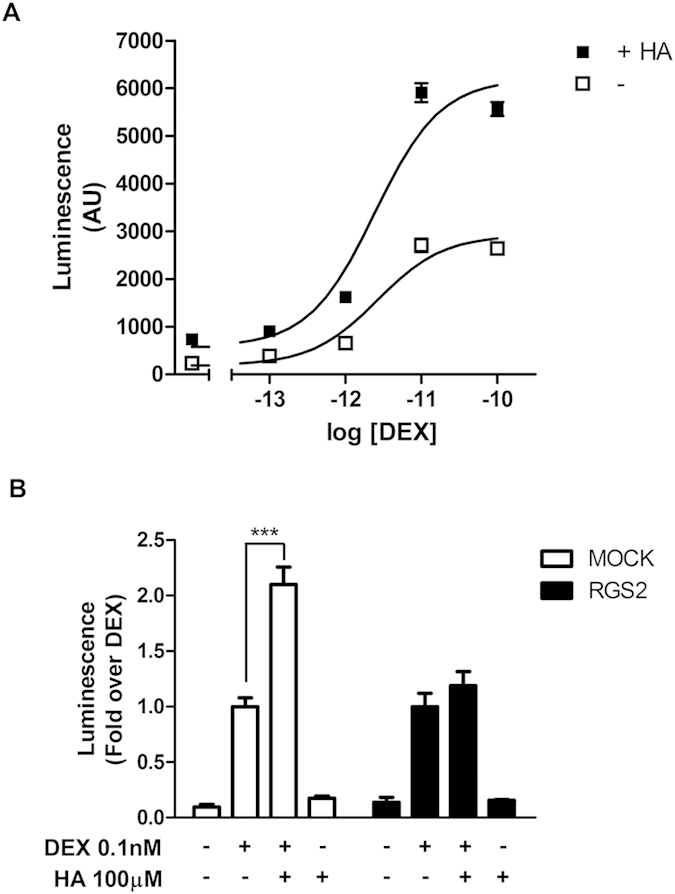
Histamine potentiates dexamethasone-induced GR activity. (**A**) HEK-293T cells co-transfected with the reporter TAT3-Luc and GR coding plasmid were treated for 10 min with 100 μM histamine (HA) or not (−), and incubated with increasing concentrations of dexamethasone (DEX). Luciferase activity was determined as described in the methods section. Curve fitting parameters are detailed in the main text. Results are mean+/−SEM of three independent experiments performed in triplicates. Error bars represent SEM. (**B**) HEK-293T cells co-transfected with TAT3-Luc, GR and H1R constructs were transfected with RGS2 or not, as indicated. Luciferase activity was determined as described in the methods section. Results are mean+/−SEM of four independent experiments performed in triplicates. ***p < 0.001.

**Figure 2:**
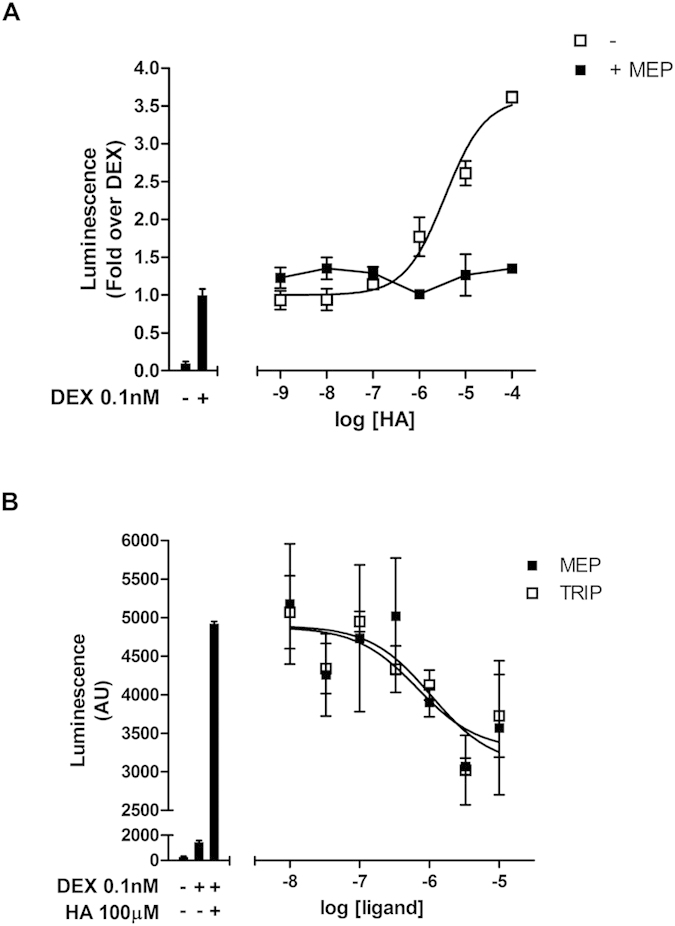
Histamine potentiation of GR activity is concentration dependent and blocked by the antihistamines mepyramine and trans-triprolidine. (**A**) HEK-293T cells were co-transfected with the reporter TAT3-Luc, GR and H1R codifing plasmids and were preincubated or not with 10 μM mepyramine (Mep) for 10 min, treated for another 10 min with increasing concentrations of histamine and incubated with 0.1 nM dexamethasone for 24 h, as indicated. Luciferase activity was determined as described in the methods section. Curve fitting parameters are detailed in the main text. Results are mean+/−SEM of three independent experiments performed in triplicates. (**B**) Co-transfected cells were preincubated with indicated concentrations of mepyramine (Mep) or trans-triprolidine (Trip), treated for another 10 min with histamine and incubated with dexamethasone for 24 h. Luciferase activity was determined as described in the methods section. Curve fitting parameters are detailed in the main text. Results are mean+/−SEM of three independent experiments performed in triplicates.

We first asked whether histamine-induced H1R signaling could influence GR-mediated transcriptional activity. Human HEK293T cells were co-transfected with a luciferase reporter plasmid under the control of a synthetic promoter regulated by the GR (TAT3-Luc) in combination with plasmids coding for GR and H1R and then cells were stimulated with dexamethasone. In this system, while 100 μM histamine induced no significant effect on luciferase activity, the addition of 100 μM histamine 10 min prior to a 24 h dexamethasone treatment induced a two-fold increment in maximal luciferase activity induced by dexamethasone alone (2923 ± 169 vs. 6201 ± 344, both expressed in luminescence arbitrary units) without affecting dexamethasone’s pEC50 (11.58 ± 0.15 vs. 11.61 ± 0.15) (Fig. 1A). The inhibition of G-protein mediated H1R signaling by co-transfection with the G-protein inhibitor RGS2 blocked this effect (Fig. 1B). Importantly, histamine did not induce any significant effect on GR mRNA levels (Supp Fig. S1). Histamine’s potentiating effect was dose dependent with a pEC50 of 5.4 ± 0.2 (Fig. 2A) and partially and dose-dependently blocked by the H1 inverse agonists mepyramine and trans-triprolidine (Fig. 2B). Histamine effect was also observed over corticosterone-induced GR activity (Supp Fig. S2). These results indicate that G-protein dependent intracellular signaling triggered by histamine-mediated activation of the H1R potentiates GR transcriptional activity induced by synthetic and natural GR agonists. However, as we aimed to elucidate the pharmacological interaction of therapeutically relevant drugs, we focused our experiments on dexamethasone-induced GR activity only.

### Role of Gα and Gβγ subunits on histamine-mediated potentiation of GR activity

**Figure 3:**
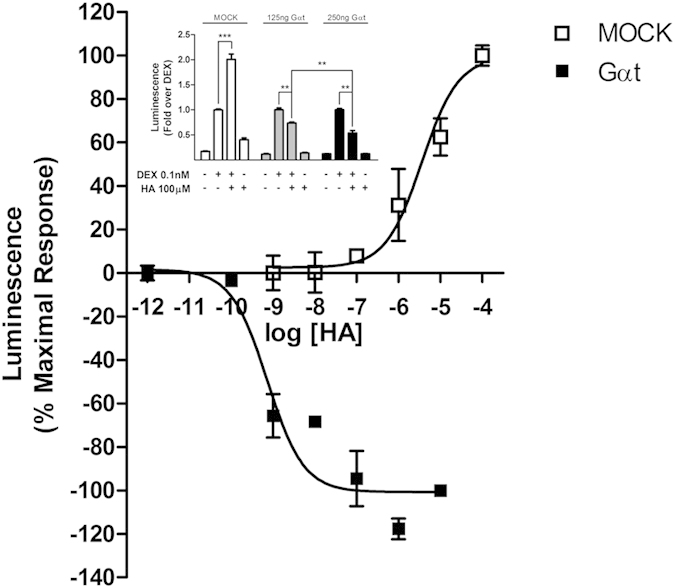
Overexpression of Gαtransducin switches histamine potentiation into inhibition of GR activity. HEK-293T cells co-transfected with TAT3-Luc, GR and H1R coding constructs were transfected or not with Gαtransducin (Gαt), as indicated. Luciferase activity was determined as described in the methods section. Results are mean+/−SEM of three independent experiments performed in triplicates. Inset: HEK-293T cells co-transfected with TAT3-Luc, GR and H1R constructs were transfected with different amounts of Gαt, and subjected to different treatments, as indicated. Luciferase activity was determined as described in the methods section. Results are mean+/−SEM of three independent experiments performed in triplicates. **p < 0.01; ***p < 0.001.

To establish which of the molecular partners participating in G-protein mediated H1R signaling were responsible of the observed potentiation of GR activity, we co-transfected HEK293T cells with the alpha subunit of the G-transducin protein (Gαt). This experimental approach is commonly used to reveal the role of Gβγ subunits on downstream effectors, based on their sequestration induced by Gαt overexpression^18^. To evaluate the appropriate transfection and expression levels of transducin, we used its known effect on carbachol-induced pERK levels^18^. For this, HEK293T cells were co-transfected with transducin or a control plasmid and a muscarinic M1 receptor coding plasmid and then stimulated with 10 μM carbachol and pERK levels were measured. As expected, transducin co-transfection precluded carbachol’s action on pERK activity (Supp Fig. S3). Remarkably, Gαt overexpression was associated with a switch in histamine’s effects from potentiation to repression of dexamethasone-induced GR activity (Fig. 3). This effect was dependent of the amount of Gαt coding plasmid transfected, suggesting that the effect of GαT is significantly augmented when plasmid concentration is increased, overall indicating a specific effect of Gαt transfection (Fig. 3 inset). These observations indicate the existence of a dual regulatory effect of H1R-mediated signaling on GR activity, a dominating potentiating effect and a secondary inhibitory effect, revealed when Gβγ subunits were sequestered. Based on these observations, we aimed to further identify signaling partners involved in both pathways.

**Figure 4:**
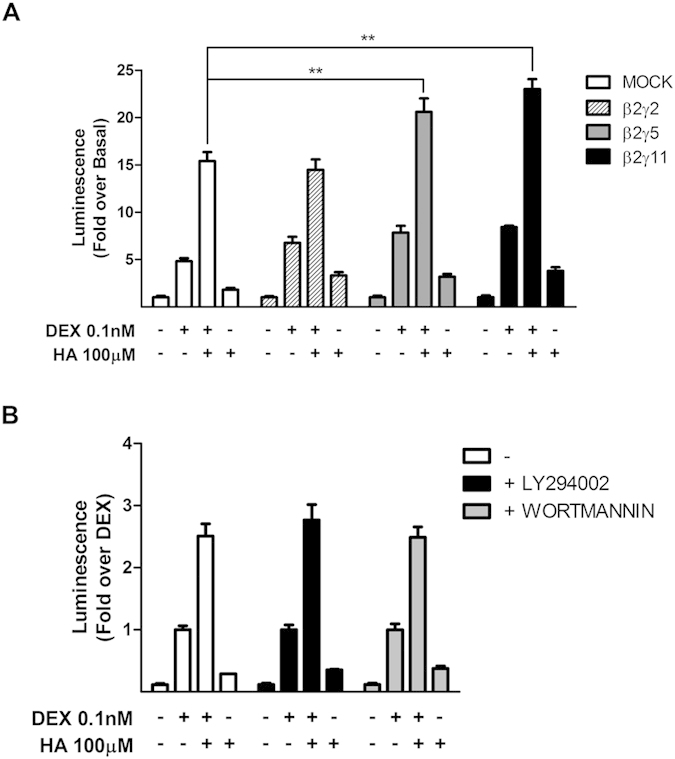
Gβ2γ5 and Gβ2γ11, but not Gβ2γ2 or PI3K are involved in histamine modulation of GR activity. (**A**) HEK-293T cells co-transfected with TAT3-Luc, GR and H1R coding constructs were transfected with β2 and γ2; β2 and γ5; β2 and γ11 or not, and subjected to indicated treatments. Luciferase activity was determined as described in the methods section. Results are mean+/−SEM of at least three independent experiments performed in triplicates. **p < 0.01. (**B**) HEK-293T cells were co-transfected with TAT3-Luc, GR and H1R coding constructs and were treated with 2 μM LY294002 or 50 nM wortmannin 30 min prior to indicated treatments. Luciferase activity was determined as described in the methods section. Results are mean+/−SEM of at least three independent experiments performed in triplicates.

To assess which Gβ and Gγ subunits may be involved in histamine’s effects on GR activity, HEK293T cells were co-transfected with Gβ2 and three different Gγ subunits, which expression was verified by fluorescence microscopy and did not affect GR expression levels (Supp Fig. S4A and S4B). We found that when the subunits combinations Gβ2γ5 and Gβ2γ11were co-expressed, histamine’s effect was increased (Fig. 4A). This potentiation of histamine’s effect was not observed with the other Gβγ combination assayed. These results suggest the existence of a mechanism involved in histamine’s potentiation of GR activity, engaging specific Gβγ subunits, including Gβ2γ5 and Gβ2γ11.

Signaling networks activated by G-proteins are very integrated and many downstream effectors have been described. PI3K plays an important role in Gβγ signaling and PI3K also modulates GR activity^15^. However, in our system, both PI3K inhibitors, LY294002 and wortmannin, had no effects on histamine modulation (Fig. 4B). Therefore we proceeded to test the effects of other possible signalling partners.

### Involvement of JNK, PLC, PKC, and Rac and Rho small G-proteins on histamine-mediated potentiation of GR activity

The c-Jun N-terminal kinase (JNK) is another kinase described both as modulator of GR activity and as effector of Gβγ dimers^19^,^20^.

**Figure 5:**
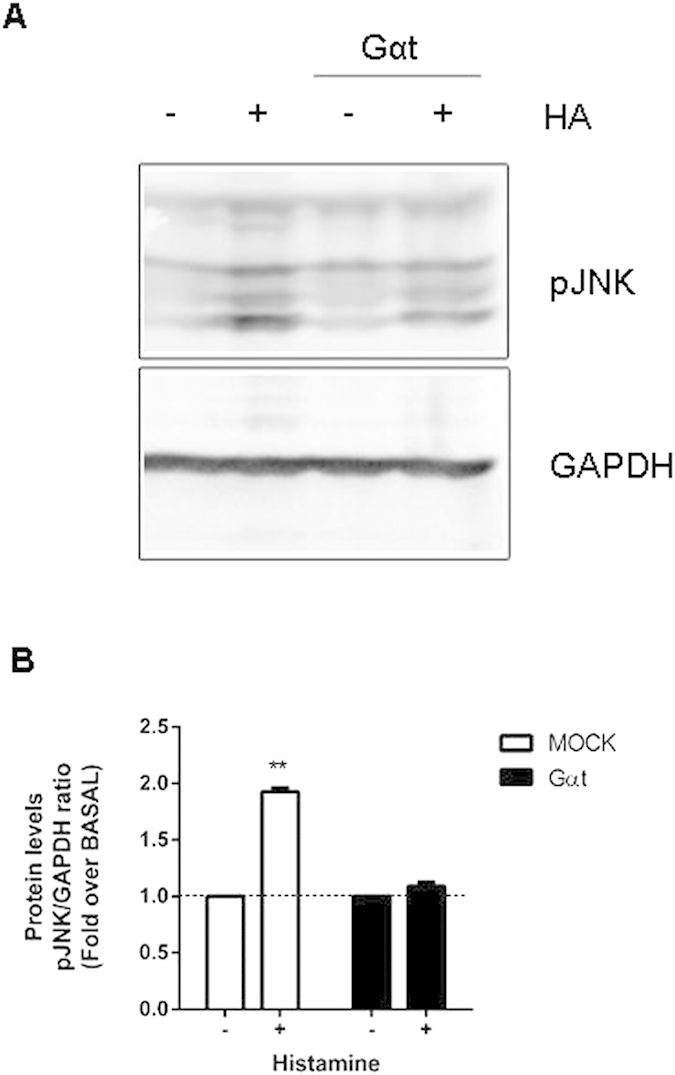
Histamine increases pJNK levels in a Gβγ dependent manner. (**A**) HEK-293T cells were transfected with a H1R coding plasmid and co-transfected or not with Gαtransducin and were subjected to 100 μM histamine treatment for 10 minutes. A cropped section of the membrane ranging from 40 to 60 kDa (upper panel) or 30 to 40 kDa (lower panel) of a representative experiment is shown. Full-length blots are provided in Supplementary Fig. 5. (**B**) Densitometric analysis was performed with ImageJ as described in the methods section. Results are mean+/−SEM of four independent experiments performed. **p < 0.01.

**Figure 6:**
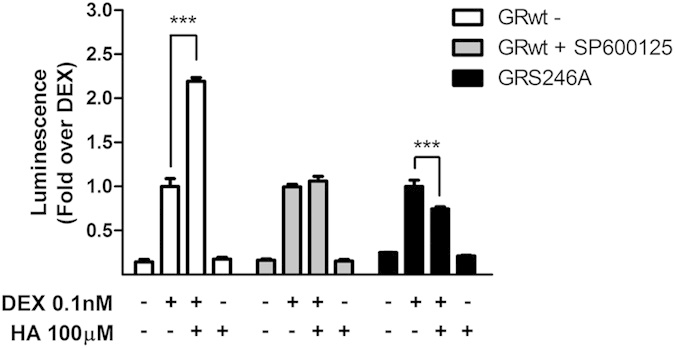
JNK impairment blocks histamine modulation of dexamethasone-induced GR activity. HEK-293T cells were co-transfected with TAT3-Luc and H1R coding constructs and were co-transfected with GR and incubated with 10 μM SP600125 for 30 min or not, or were co-transfected with GR-S246A, as indicated, and then subjected to treatments. Luciferase activity was determined as described in the methods section. Results are mean+/−SEM of three independent experiments performed in triplicates. ***p < 0.001.

In our system, histamine increased phospho-JNK levels and histamine’s effect was abolished by overexpression of Gαt, suggesting the involvement of Gβγ subunits (Fig. 5 and Supp Fig. S5). This makes JNK a suitable candidate participating in histamine’s potentiation of GR transcriptional activity. Consistently, the JNK inhibitor SP600125 completely abolished histamine’s potentiation of GR response to dexamethasone (Fig. 6). Moreover, overexpression of a JNK phosphorylation-deficient mutant, GR-S246A, showed that histamine’s effect was dependent on JNK-mediated phosphorylation of the GR (Fig. 6). These results indicate that histamine-induced activation of Jun kinase is crucially involved in its potentiating effect on GR transcriptional activity. Dexamethasone induces GR phosphorylation at S246, with diverse effects on its transcriptional activity^21^,^22^. In our hands, the GR mutant S246A showed the same efficacy in the gene-reporter assay as the wild type GR, indicating that dexamethasone-induced GR phosphorylation at S246 had little, if any, effect on its transcriptional activity in our system (Fig. 6). These observations suggest that the minor effects we detect in transcriptional activity may be related to intrinsic properties in the cell lines we used in our studies and/or with the sensitivity of our detection method or other technical limitations.

Previous observations have shown that after JNK activation by overexpression of its direct activator MKK7, JNK-mediated GR phosphorylation results in a decrease of GR-dependent transcriptional activity^20^,^23^. However, these effects are dependent on the promoter-context, since JNK activation has opposite effects on TAT3- and MMTV-driven transcriptional activity^24^. We assessed histamine’s effect on MMTV-driven transcriptional activity induced by dexamethasone. In our hands, histamine also potentiated dexamethasone-induced expression of a MMTV-driven luciferase reporter, and this effect was hampered by overexpression of GR-S246A, suggesting again involvement of JNK. To evaluate the possible influence of the cellular context, we also performed experiments in HeLa cells, which were originally used to characterize the effect of JNK on GR activity. Again, histamine potentiated GR activity on both TAT3- and MMTV-driven luciferase reporter systems (Supp Fig. S6). Together, these results suggest that histamine-induced JNK activation may take place by alternative, yet uncharacterized intracellular pathways that do not engage MKK7 directly^25^,^26^. Overall, previous work and ours show the complexity of the impact of S246 phosphorylation on GR activity. However, the exact characterization and disambiguation of this complex effects escapes the aim of our work at this point.

**Figure 7:**
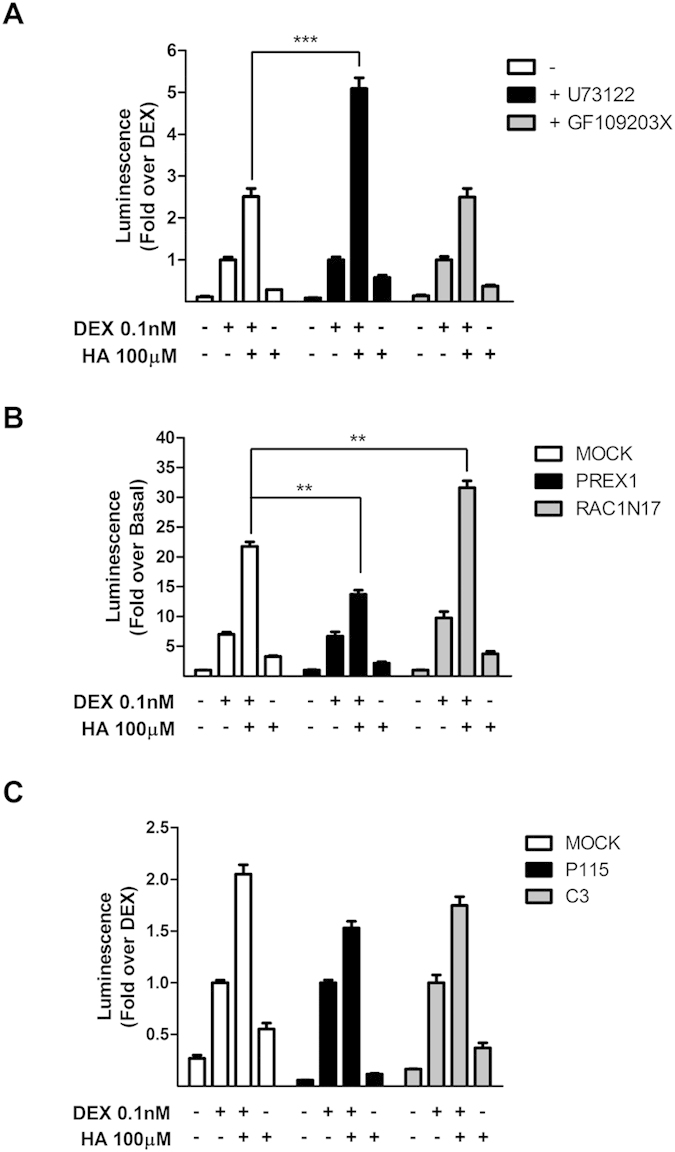
PLC and Rac, but not PKC or Rho are involved in histamine modulation of dexamethasone-induced GR activity. (**A**) HEK-293 T cells were co-transfected with TAT3-Luc, GR, and H1R coding constructs and were incubated for 30 min with 10 μM U73122 (PLC inhibitor) or 10 μM GF109203X (PKC inhibitor), and then subjected to indicated treatments. (**B**) HEK-293 T cells were co-transfected with TAT3-Luc, GR, and H1R coding constructs and were co-transfected with Rac GEF Prex1 or with the dominant negative RacN17 and then subjected to indicated treatments. (**C**) HEK-293T cells were co-transfected with TAT3-Luc, GR, and H1R coding constructs and were co-transfected with Rho GEF P115 or with the Rho GAP C3 toxin, and then subjected to indicated treatments. Luciferase activity was determined as described in the methods section. Results are mean+/−SEM of three independent experiments performed in triplicates. **p < 0.01; ***p < 0.001.

Finally, we tried to characterize signaling partners involved in the inhibitory pathway, focusing on Gα. Using the specific inhibitors U73122 and GF109203X, we conclude that PLC but not PKC may be implicated in histamine’s inhibitory effect on GR transcriptional activity (Fig. 7A). Small G-proteins, such as Rac and Rho, have been characterized as downstream effectors of H1R signaling^27^. To evaluate their possible role in H1R modulation of GR activity, we used Rac and Rho GEFs Prex1 and p115, as activators, and Rac N17 and C3 exoenzyme, as Rac and Rho inhibitors, respectively. We found that Rac activation reduced histamine’s potentiation of GR activity. Consistently, the dominant negative mutant Rac N17 enhanced histamine’s effect (Fig 7B). Conversely, expression of p115 Rho-GEF or C3 exoenzyme had no detectable effect on histamine’s potentiation of GR activity (Fig. 7C), indicating that Rho does not participate in this process.

### Effect of antihistamines on glucocorticoid-induced gene transactivation and transrepression

**Figure 8:**
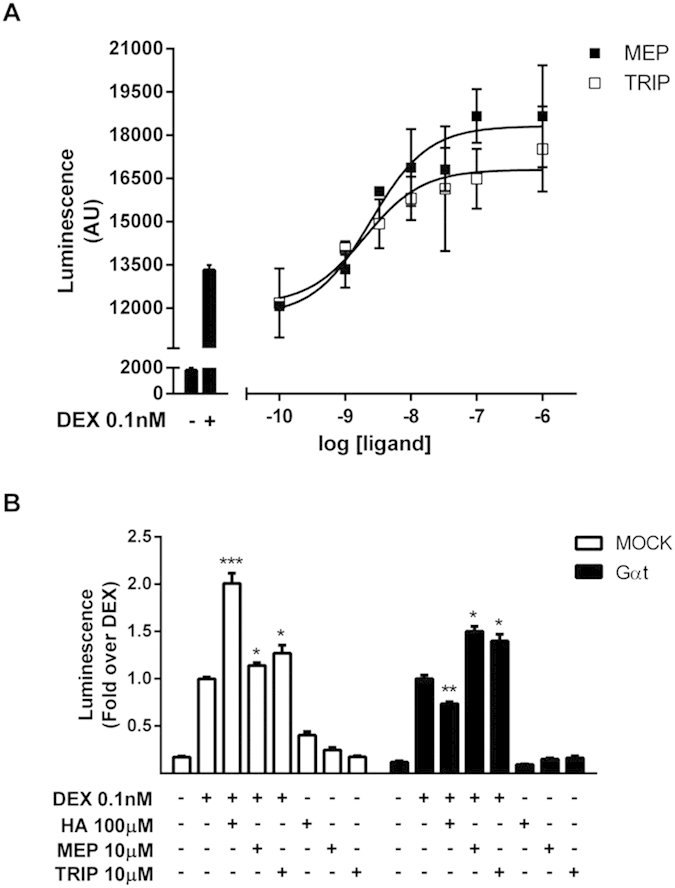
Antihistamines potentiate dexamethasone-induced GR activity and the overexpression of Gαtransducin does not inhibits their effects. (**A**) HEK-293T cells co-transfected with the reporter TAT3-Luc, GR and H1R coding plasmids were preincubated for 10 min with increasing concentrations of mepyramine (Mep) or trans-triprolidine (Trip) and incubated with 0.1 nM dexamethasone for 24 h. (**B**) HEK-293T cells were co-transfected with TAT3-Luc, GR and H1R coding constructs and were transfected or not with Gαtransducin, as indicated, and subjected to treatments. Luciferase activity was determined as described in the methods section. Results are mean+/−SEM of three independent experiments performed in triplicates. *p < 0.05; **p < 0.01; ***p < 0.001.

To further evaluate the possible implications of our observations for a clinical scenario involving the pharmacological association of a GR agonist and an antihistamine, we studied the effect of two clinically relevant H1 inverse agonists, mepyramine and trans-triprolidine, on dexamethasone-induced GR transcriptional activity. We found that both antihistamines dose-dependently potentiated dexamethasone-induced transcriptional activity (from 11935 ± 1068 to 18317 ± 561 for mepyramine and to 16806 ± 632 for trans-triprolidine; Fig. 8A). This potentiating effect induced by the H1R inverse agonists on GR activity could be due to a decrease in Gα activity and its downstream inhibitory effectors or to an increase in a Gβγ-mediated pathway. However, the stimulatory activity induced by both inverse agonists was not affected by Gαt expression, ruling out the involvement of Gβγ subunits (Fig. 8B).

**Figure 9:**
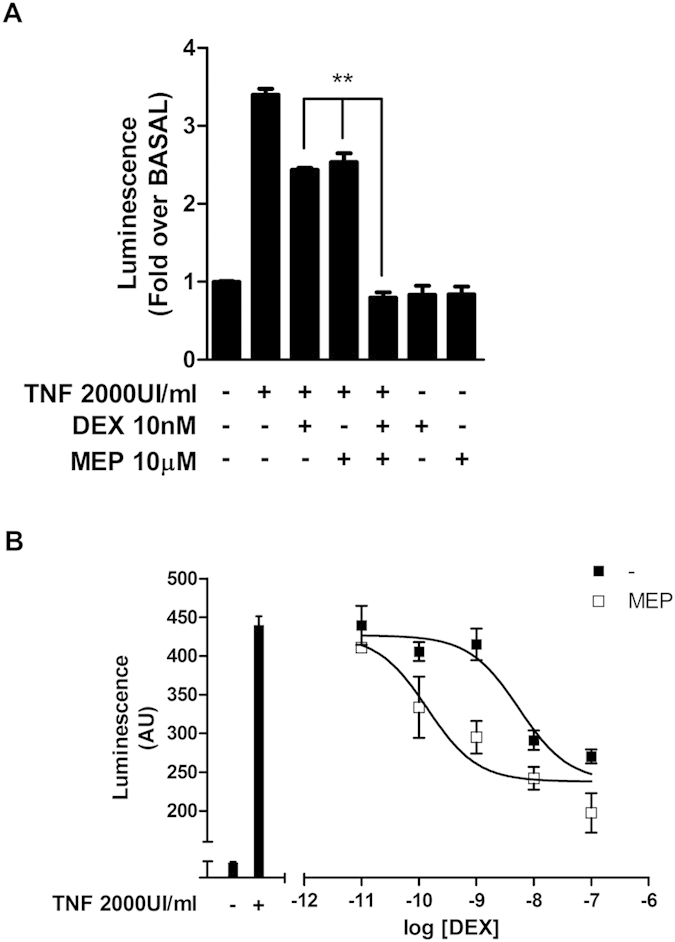
Mepiramine potentiates dexamethasone-induced GR transcriptional activity in a transrepression gene-reporter system. (**A**) HEK-293T cells were co-transfected with IL6-Luc, GR and H1R coding constructs and were incubated with 2000 UI/ml TNFα for 4 h, and exposed to 10 μM mepiramine for 10 min and to 10 nM dexamethasone for 24 h, as indicated. (**B**) HEK-293T cells were co-transfected with IL6-Luc, GR and H1R coding constructs and were incubated with 2000 UI/ml TNFα for 4 h, and then exposed to 10 μM mepiramine for 10 min and incubated with increasing concentrations of dexamethasone for 24 h. Curve fitting parameters are detailed in the main text. Luciferase activity was determined as described in the methods section. Results are mean+/−SEM of three independent experiments performed in triplicates. *p < 0.05; **p < 0.01; ***p < 0.001.

Transrepression mechanisms are relevant for the effectiveness of glucocorticoids as anti-inflammatory agents. Considering this, we tried to elucidate if the potentiation induced by H1R inverse agonists on GR activity was conserved on a IL6 promoter-driven luciferase reporter, typically transrepressed by dexamethasone. Luciferase expression was induced by pre-exposing cells to TNFα and then, the effect of GR activation was measured. Dexamethasone and mepyramine induced a 30% and 25% decrease in luciferase, respectively. Remarkably, when cells were co-incubated with both ligands, a synergistic effect was observed resulting in a 75% reduction in luciferase signal (Fig. 9A). This synergism was confirmed by a left-shift on dexamethasone concentration-response curve (13.74 ± 0.24 vs. 12.14 ± 0.23) (Fig. 9B).

### Effect of H1R inverse agonists on endogenous inflammation-related gene expression induced by GR activation

Next, we aimed to assess whether H1R-mediated potentiation of dexamethasone-induced gene expression could be replicated in a pathophysiologically relevant cell system, where GR agonists and antihistamines are expected to interact. We used A549 alveolar epithelial cells, as a model of alveolar reactivity to noxious stimuli and thus a suitable *in-vitro* model reflecting aspects of pulmonary inflammation^28^,^29^, and U937 promonocytic cells, a model of skin sensitization^30^,^31^. The H1R is expressed in U937 promonocytic cells^32^,^33^, however we could not find any report of the expression of the H1R in the A549 cells. Therefore, we tested the presence of a functional H1R in A549 cells by measuring the modulation of intracellular calcium levels. 100 μM histamine was able to produce an intracellular calcium spike that was specifically blocked by preincubation with mepyramine, demonstrating H1R functionality (Supp Fig. S7). Using these two cellular systems, we assayed the expression of three primary genes transactivated by the GR^34^: GILZ (glucocorticoid induced leucine zipper), THBD (thrombomodulin) and SLC19A2 (thiamine transporter 1 or solute carrier family 19 member 2); and the typically GR-transrepressed proinflammatory gene COX-2 (cyclooxigenase-2)^35^.

**Figure 10:**
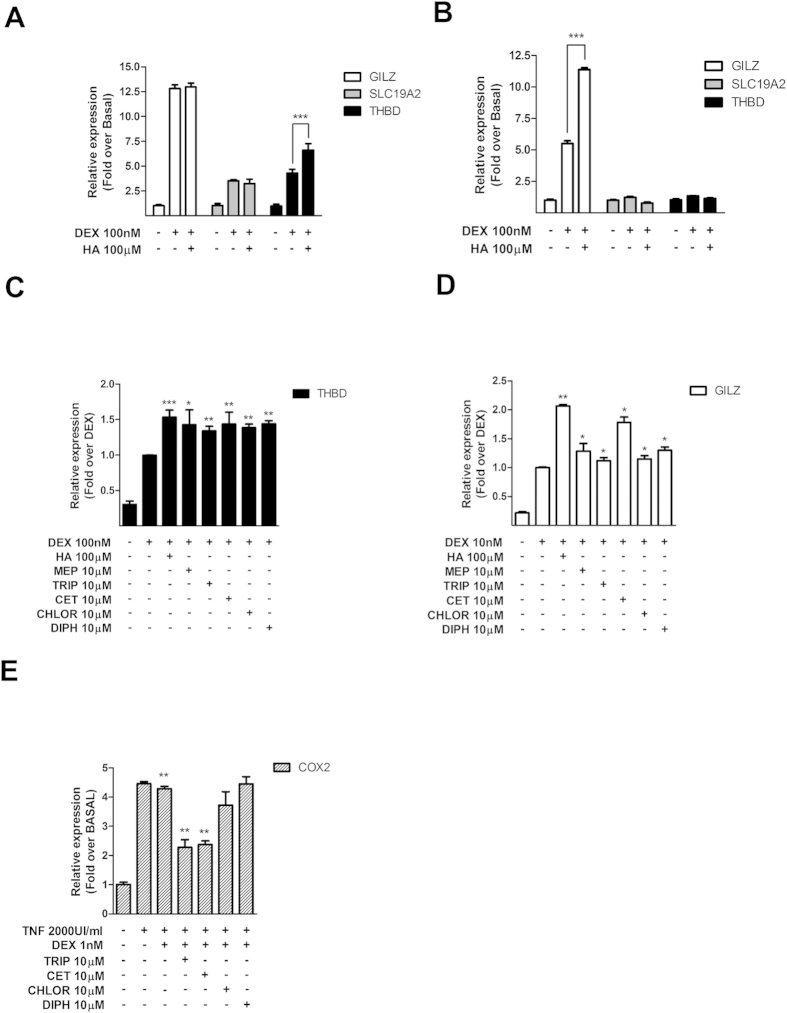
Histamine and antihistamines modulate dexamethasone-induced expression of endogenous GR-target genes. (**A, C**) A549 cells and (**B, D**) U937 cells were incubated for 10 min with 100 μM histamine (HA), 10 μM mepyramine (MEP), 10 μM trans-triprolidine (TRIP), 10 μM cetirizine (CET), 10 μM chlorpheniramine (CHLOR), or 10 μM diphenhydramine (DIPH), as indicated, and then treated with dexamethasone (DEX) for 3 h. **(E)** A549 cells were incubated with TNFα for 4 h, exposed to 10 μM trans-triprolidine (TRIP), 10 μM cetirizine (CET), 10 μM chlorpheniramine (CHLOR), or 10 μM diphenhydramine (DIPH), as indicated, and treated with dexamethasone (DEX) for 3 h. GILZ, SLC19A2, THBD, and COX-2 mRNA levels were quantified by qPCR as described in the methods section. Results are mean+/−SEM of at least three independent experiments performed in triplicates. *p < 0.05; **p < 0.01; ***p < 0.001.

In A549 cells, dexamethasone increased the expression of the three transactivated genes. However, histamine potentiated only THBD expression (Fig. 10A). In U937 cells, only GILZ expression was induced by dexamethasone and this effect was potentiated by histamine (Fig. 10B). These observations suggest cell-type and gene-specific effects consistent with previous reports of GR cell type- and gene-specific actions^36^, and indicate that the results obtained in gene reporter assays can not be simply extrapolated to all endogenous GR-responsive genes.

However, pre-exposure of A549 and U937 cells to each antihistamine tested, mepyramine, trans-triprolidine, cetirizine, chlorpheniramine, or diphenhydramine induced, in all cases, a potentiation of dexamethasone-induced THBD and GILZ expression (Fig. 10C, D), confirming that the results obtained with luciferase could be of clinical and pharmacological interest. The regulation of COX-2 expression could not be evaluated in U937 cells because we were unable to measure any significant effect of TNFα on COX-2 expression in this cell line (Supp Fig. S8). However, although dexamethasone by itself only minutely decreased TNFα-induced COX-2 expression in A549 cells, pretreatment with trans-triprolidine and cetirizine, but not chlorpheniramine or diphenhydramine, potentiated dexamethasone-induced inhibition of COX-2 expression (Fig. 10E). These observations indicate that some H1R inverse agonists are able to modulate GR-induced transrepression, as well. In all cases, cetirizine showed the highest efficacy in potentiating dexamethasone-induced GR activity. Our observations are thus consistent with previous work showing that cetirizine is the most efficacious antihistamine available^37^.

## Discussion

In a number of clinical situations related to inflammatory disease, ligands of both H1R and GR are co-administered^37^,^38^. However, this drug association has no clear rationale and has arisen from the clinical practice, based on the assumption that there should be an intrinsic benefit in their co-administration, due to their anti-inflammatory effects. In this work we studied the consequences of co-treatment with clinically relevant antihistamines and glucocorticoids at the molecular level in several *in-vitro* cellular systems. Using these simple systems, we characterized for the first time a synergistic effect between the H1R and the GR, with particular focus on GR-induced transcriptional activity.

Our results suggest that activation of the H1R triggers a complex dual regulatory mechanism on GR activity, involving both Gαq and Gβγ subunits. Whereas Gαq has an inhibitory effect via a PLC-RAC-mediated pathway, Gβγ enhances GR activity via JNK. Paradigmatically, when an agonist binds to a GPCR, it causes the activation of the trimeric G-protein and its subsequent dissociation into Gα and Gβγ subunits, turning on two downstream pathways. Within this framework, both pathways may be equally activated. Thus, when agonists bind to, and activate the H1R, Gαq and Gβγ are simultaneously released. Under these circumstances, the Gβγ pathway prevails, resulting in an overall H1R-mediated potentiation of GR activity. Importantly, other GPCRs, such as the β2-adrenergic, somatostatin and melatonin receptors, have been shown to modulate GR activity via pathways that, at least partially, engage the action of Gβγ subunits^14^,^15^,^16^, suggesting this may be a widespread pathway for GPCR/GR regulatory interactions.

On the other hand, when antihistamines bind to, and inactivate the H1R, a potentiating effect on GR activity is observed too, which in this case can be attributed to the inactivation of the Gαq-PLC-RAC inhibitory pathway. Thus, the initially paradoxical observation that the natural full agonist as well as the inverse agonists potentiate GR transcriptional activity can be explained by a decrease in the Gαq-mediated inhibition of GR activity induced by inverse agonists.

Binding of the GR to simple GREs induces epigenetic mechanisms such as histone acetylation and chromatin remodeling and recruits RNA polymerase II by interactions with coactivator molecules, which in many cases, have acetyltransferase activity and are part of the cellular epigenetic machinery, finally resulting in transactivation of GC target genes^6^. Therefore, one intriguing possibility is that some of the signaling pathways activated by H1R activation could result in ‘priming’ of GR activity to pro-inflammatory genes^38^, by recruiting (epigenetic) cofactors to specific GR target sites. However, this tempting hypothesis escapes the direct aim of this study and requires experimental validation.

Relevant cell types where the signaling convergence identified in our work may be relevant should express both H1R and GR, such as endothelial cells, dendritic cells, monocytes, neutrophils, T and B cells and microglia^39^,^40^. The existence of these cell types co-expressing both receptors suggests that our findings may have implications for regulation of inflammation in several systems, such as lung, skin and brain. However, this point requires further experimental validation.

Although our preliminary characterization of the signaling events triggered by the co-administration of antihistamines and glucocorticoids is restricted to simple cellular systems *in vitro*, granting further *in vivo* validation of the mechanisms described herein, our observations may have implications for the understanding of the cellular mechanisms triggered by the widespread clinical use of this drug association. Of particular concern, our results indicate that the co-administration of an antihistamine and a glucocorticoid agonist may result in a potentiation of the transactivation and transrepression of some genes key in the inflammatory response. At the same time, transactivation of GR-target genes which may be involved in GC’s side effects at pharmacological concentrations could be induced, indicating that adverse effect may be potentiated *in vivo* as well. This possibility suggests the need of a careful (re)evaluation of the common coadministration of antihistamines and glucocorticoids in the treatment of inflammatory conditions. Some illustrative examples, such as are the treatment of AR and AD, suggest that our observations could have clinical implications. A study from 2011 showed that the combination of both drugs is the most widely-used option in routine clinical practice to treat all types of AR^2^, and three patents have been recently granted to formulations containing the antihistamine azelastine with the synthetic corticosteroids mometasone furoate, ciclesonide and fluticasone propionate^3^. Likewise, concerning AD, the synergistic effects of glucocorticoids and antihistamines have been evaluated in an animal model, where olopatadine potentiated the inhibitory effect of prednisolone on the relief of the inflammatory symptoms leading to the conclusion that this drug combination is useful to treat AD, although the mechanism underlying the synergism was not investigated^41^.

Different approaches have been used to improve the beneficial/undesired effect ratio in the clinical administration of glucocorticoids, going from chemical optimization to development of selective glucocorticoid receptor agonists (SEGRAs) assuming that ligands that only induce gene transrepression but not transactivation should have a better therapeutic profile. The add-on therapies present another alternative to control the side effects associated with corticosteroid administration by combining it with another aintiinflamatory drug with a different mechanism of action, allowing a reduction in corticosteroid dose and a concomitant reduction in adverse effects, since these are more common in patients receiving high corticosteroid doses. In this approach, corticoids are combined for example, with β2-adrenoreceptor agonists, teophylline or anti-leukotrienes^42^.

In this regard, combination therapy of GC and antihistamines can represent a clinically relevant option. Although not experimentally tested in this work, co-treatment with a glucocorticoid and an antihistamine might allow reductions in the dose of the glucocorticoid *in vivo*, which could have beneficial effects in the clinic. Suggestively, the potentiating effect of antihistamines on GR transcriptional activity described here with two GRE-dependent luciferase gene reporter systems and endogenous GR-responsive and inflammation-related genes, resulted in a reduction of the doses of dexamethasone needed to achieve the same biological effect. Although some of these effects were cell type and gene-specific, our results indicate that under certain circumstances the coadministration of antihistamines could result in the reduction of the dexamethasone doses need to reach anti-inflammatory effects. Supporting this hypothesis it has been shown that the antihistamine azelastine can reduce the frequency of administration of inhaled corticosteroids without loss of pulmonary function on a clinical trial on patients with chronic bronchial asthma^43^.

In conclusion, the molecular characterization of the interactions between intracellular signaling cascades triggered by activation of the H1R by histamine and its inverse agonists and GR- mediated transcriptional activity presented in this work provides a starting point for understanding and re-evaluating the use of antihistamines as add-on drugs in glucocorticoid-mediated anti-inflammatory therapies. Further characterization and validation of this interaction at the molecular level, its possible relevance and its pharmacokinetic and pharmacodynamics properties *in vivo*, will help to improve commonly used combination therapies.

## Materials and Methods

### Materials

DMEM medium, antibiotics, phosphate-buffered saline (PBS), bovine serum albumin (BSA), histamine dihydrochloride, wortmannin, SP600125, U73122, dexamethasone, cetirizine, chlorpheniramine and diphenhydramine were obtained from Sigma. LY294002, mepyramine maleate, trans-triprolidine and GF109203X were from Tocris Cookson Inc. (Ballwin, MO). Fetal bovine serum (FBS) was purchased from Natocor. All other chemicals were of analytical grade and obtained from standard sources.

### Plasmid Constructions

pRSV-GR was cloned by Dr. Keith Yamamoto^44^. pCEFL-H1R and TAT3-Luc were previously generated in our laboratory^12^,^45^. MMTV-Luc was provided by Dr. A Pecci (IFIBYNE, CONICET, UBA) and IL6-Luc was a gift from Prof. Dr. Karolien De Bosscher (VIB Department of Medical Protein Research, University of Gent, Belgium). pcDNA3-C3 was granted by Dr. Omar Coso (IFIBYNE, CONICET, UBA). pCMV-Myc-PRex1, pcDNA3-RGS2 and pcDNA3-p115 were kindly provided by Dr. Marcelo Kazanietz (University of Pennsylvania School of Medicine, Philadelphia), pEGFP-C1-Gβ2 and pEGFP-C1-Gγ2 were kindly provided by Dr. Tomoshige Kino (DeCherney Lab—Section on Implantation and Oocyte Physiology, National Institutes of Health, Bethesda), pENTR-YFP-Gγ5 and pcDNA3-1-YFP-Gγ11 were a kind gift from Dr. N Gautam (Anesthesiology and Genetics Dpt, Washington University School of Medicine, St. Louis). pCEFL-Gα transducin was a kind gift from Dr. Silvio Gutkind (Oral and Pharyngeal Cancer Branch, National Institutes of Health, Bethesda), pEYFP-N1-M1R was provided by Dr. JC Goin (CEFYBO, CONICET, UBA), pcDNA3-HA-GR-S246A was a gift from Dr. Marija Krstic-Demonacos (Molecular Medicine. University of Salford, Manchester, UK), and pcDNA3-RacN17 was kindly provided by Dr. Heidi Welch (Babraham Institute, Cambridge).

### Cell culture

HEK293T (human embryonic kidney), HeLa (cervical cancer), and A549 (human pulmonary) cells were cultured in Dulbecco’s modified Eagle’s medium (DMEM). U937 (human promonocytic) cells were cultured in RPMI 1640 medium. All mediums were supplemented with 10% fetal calf serum and 5 μg/ml gentamicin and cells were incubated at 37 °C in humidified atmosphere containing 5% CO_2_.

### Transfection and reporter gene assays

HEK293T or HeLa cells seeded on 24-well plates were co-transfected using the K2 Transfection System (Biontex, Munich, Germany) with the TAT3-Luc, MMTV-Luc or IL6-Luc luciferase reporter plasmids and pRSV-GR according to the manufacturer’s instructions. In some experiments, cells were also co-transfected with the plasmid constructs indicated in the corresponding figure or an empty vector to maintain an equal amount of total DNA. After 4h, cells were seeded in 96-well plates, and 24 h later cells were starved overnight and then stimulated with diverse agents. After a kinetic assessment, luciferase activity was measured at the optimal time of 24 h later using the Steady-Glo Luciferase Assay System according to the manufacturer’s instructions (Promega Biosciences Inc. San Luis Obispo, CA, USA) using a FlexStation 3 Multi-Mode Microplate Reader (Molecular Devices, LLC). Experimental reporter activity was normalized to control activity. No differences were observed in results normalized to renilla-luc or to protein expression levels.

### RT-PCR and Quantitative real-time PCR

Total RNA was isolated from A549 or U937 cells using Quick-Zol reagent (Kalium Technologies) following the manufacturer’s instructions. For the first-strand cDNA synthesis, 1 μg of total RNA was reverse-transcribed using the High Capacity cDNA Reverse Transcription kit (AB) with random primers. Quantitative real-time PCR (qPCR) was performed in triplicate on the Rotor Gene Q cycler (Qiagen) using the resulting cDNA, the HOT FIREPol EvaGreen qPCR Mix Plus (Solis Biodyne) for product detection, and the following primers: human GILZ (glucocorticoid induced leucine zipper; NM_001015881.1) forward, 5′-AATGCGGCCACGGATG-3′ and reverse, 5′-GGACTTCACGTTTCAGTGGACA-3′; THBD (thrombomodulin; NM_000361.2) forward, 5′-GACCTTCCTCAATGCCAGT-3′ and reverse, 5′- CCGTTCAGTAGCAAGGAAATG-3′; SLC19A2 (thiamine transporter 1 or solute carrier family19 member 2; NM_006996.1) forward, 5′-TTCTCTGCTGGTCTGTGTGG-3′ and reverse, 5′-AGCGAGAAGGCATCACTTTC-3′; COX-2 (cyclooxigenase-2; NM_000963.1) forward 5′-TTCAAATGAGATTGTGGGAAAATTGCT-3′ and reverse 5´-AGATCATCTCTGCCTGAGTATCTT-3´, GR (glucocorticoid receptor; NP_000167.1) forward 5′-TACCCTGCATGTACGACCAA-3′ and reverse 5′-TCCTTCCCTCTTGACAATGG-3′; and human β-Actin (βAct) forward, 5′-GGACTTCGAGCAAGAGATGG-3′ and reverse 5′-AGCACTGTGTTGGCGTACAG-3′. The cDNA was amplified by 45 cycles of denaturing (10 s at 95 °C), annealing (10 s at 60 °C), and extension (10 s at 72 °C) steps. The specificity of each primer set was monitored by analyzing the dissociation curve, and the relative GILZ, THBD or SLC19A2 mRNA quantification was performed using the comparative ΔΔCt method using β-Actin as the housekeeping gene.

### Western Blot Assay

Cells were lysed in 50 mM Tris-HCl, pH 6.8, 2% SDS, 100 mM 2-mercaptoethanol, 10% glycerol, and 0.05% bromophenol blue and sonicated to shear DNA. Total cell lysates were resolved by 10% SDS-PAGE for pJNK detection, blotted, and incubated with 1 μg/ml rabbit polyclonal anti-pJNK or anti-pERK (Santa Cruz Biotechnology) in PBS containing 0.05% Tween 20. All subsequent washes were performed with the same buffer. Reactivity was developed using an anti-rabbit polyclonal antibody linked to horseradish peroxidase (Santa Cruz Biotechnology) and enhanced chemiluminescence reagents following the manufacturer’s instructions (Amersham Biosciences). Thereafter, membranes were stripped with stripping buffer before being reprobed with anti-GAPDH (Santa Cruz Biotechnology) to ensure equal loadings.

### Data Analysis

Statistical analysis was performed through GraphPad Prism 5.0 (GraphPAD Software for Science, San Diego, CA, USA). Results are expressed as mean ± SEM. Parametric statistical analysis was performed using one- or two-way ANOVA, followed by Bonferroni *post hoc* multiple comparisons. Differences were considered significant at p < 0.05.

## Additional Information

**How to cite this article**: Daniel, Z. C. *et al.* Effects of histamine H1 receptor signaling on glucocorticoid receptor activity. Role of canonical and non-canonical pathways. *Sci. Rep.* **5**, 17476; doi: 10.1038/srep17476 (2015).

## Supplementary Material

Supplementary Information
