# Supplementary material for: Effects of histamine H1 receptor signaling on glucocorticoid receptor activity. Role of canonical and non-canonical pathways
Source: Sci Rep. 2015 Dec 4;5:17476. doi: 10.1038/srep17476 (PMC4669453; doi:10.1038/srep17476)
Supplement: Supplementary Information [file srep17476-s1.pdf]

Supplemetary results corresponding to: Effects of histamine H1 receptor signaling on glucocorticoid receptor activity. Role of canonical and non-canonical pathways.

Zappia Carlos Daniel; Granja-Galeano Gina; Fernández Natalia; Shayo Carina; Davio Carlos; Fitzsimons Carlos P; Monczor Federico.

Histamine does not regulate glucocorticoid receptor mRNA levels.

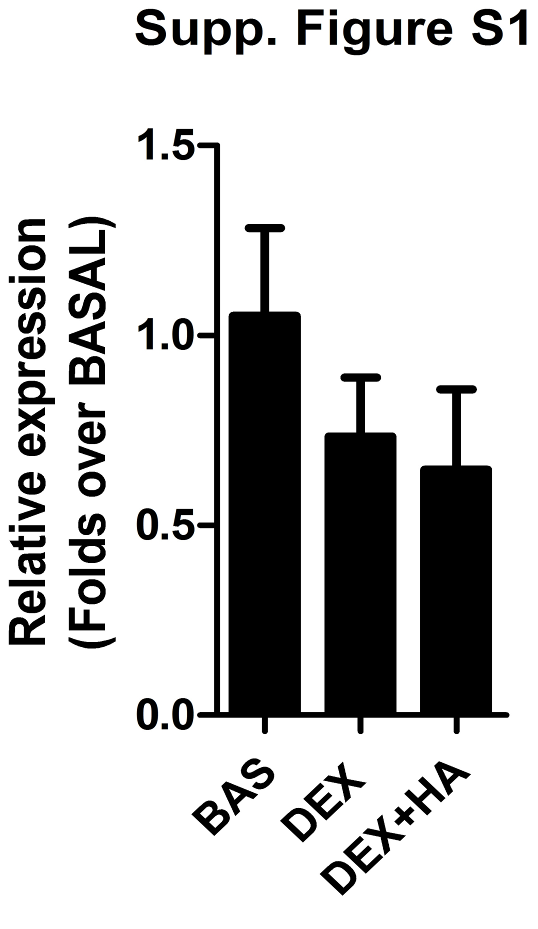

Supplementary figure 1. Hek293T cells transfected with pRSV-GR were incubated for 10 min with 100µM histamine (HA) and then treated with dexamethasone (DEX) for 24 h. GR mRNA levels were quantified by qPCR as indicated in methodology section.

Histamine potentiates costicosterone-induced GR activity.

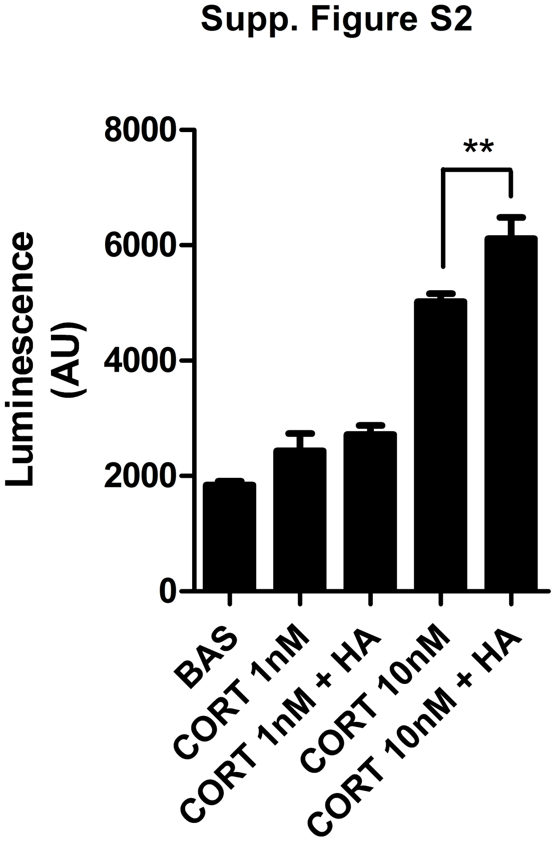

Supplementary figure 2. HEK-293T cells co-transfected with the reporter TAT3-Luc and GR codifying plasmid were treated for 10 min with 100μM histamine (HA) and corticosterone (Cort) for 24 h. Luciferase activity was determined as described in methods section. Results are mean+/-SEM of four independent experiments performed in triplicates. **p<0.01

Gαtransducin prevents carbachol-mediated increase in p-ERK levels.

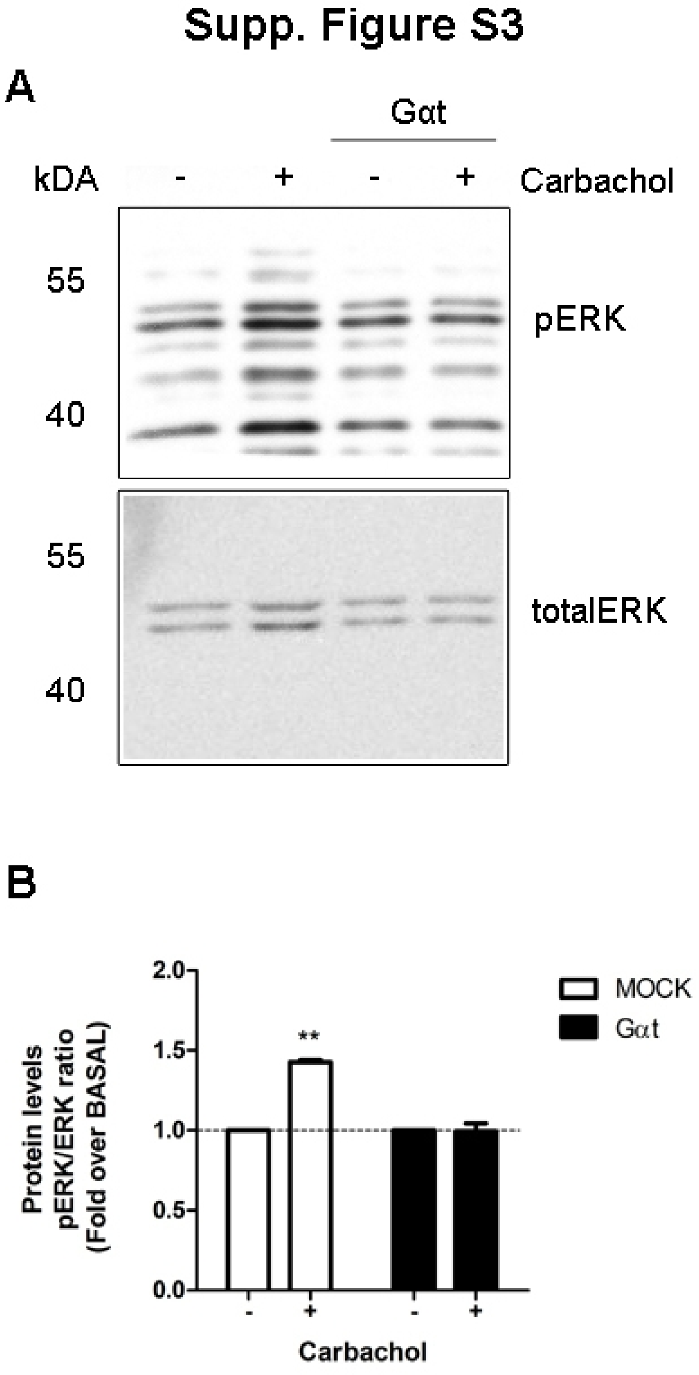

Supplementary figure 3. (A). HEK-293T cells transfected with the muscarinic M1 receptor codifying plasmid and co-transfected or not with Gαtransducin were subjected to 10 µM carbachol treatment for the 10 minutes. A membrane of a representative experiment is shown. **(B).** Densitometric analysis was performed with ImageJ as indicated in methodology section. Results are mean+/-SEM of four independent experiments performed. ** p<0.01.

Expression of GFP-Gβ2, GFP-Gγ2, YFP-Gγ5, and YFP-Gγ11 determined by fluorescence microscopy and its effect on glucocorticoid receptor mRNA levels.

HEK293T cells were transfected with the indicated plasmids as described above. After 4h cells were seeded on poly-L-lysine-coated cover slides and cultured for 48h. Then they were fixed with 4% PFA and subsequently mounted on glass slides. Microscopic images were digitally captured with a Nikon Eclipse E400 microscope (Nikon, Tokyo, Japan; illumination: 6 V halogen lamp, 20 W, equipped with a stabilized light source) via a Sony SSC-DC50 camera. Quantitative PCR was developed as stated on methodology section.

Supp. Figure S4

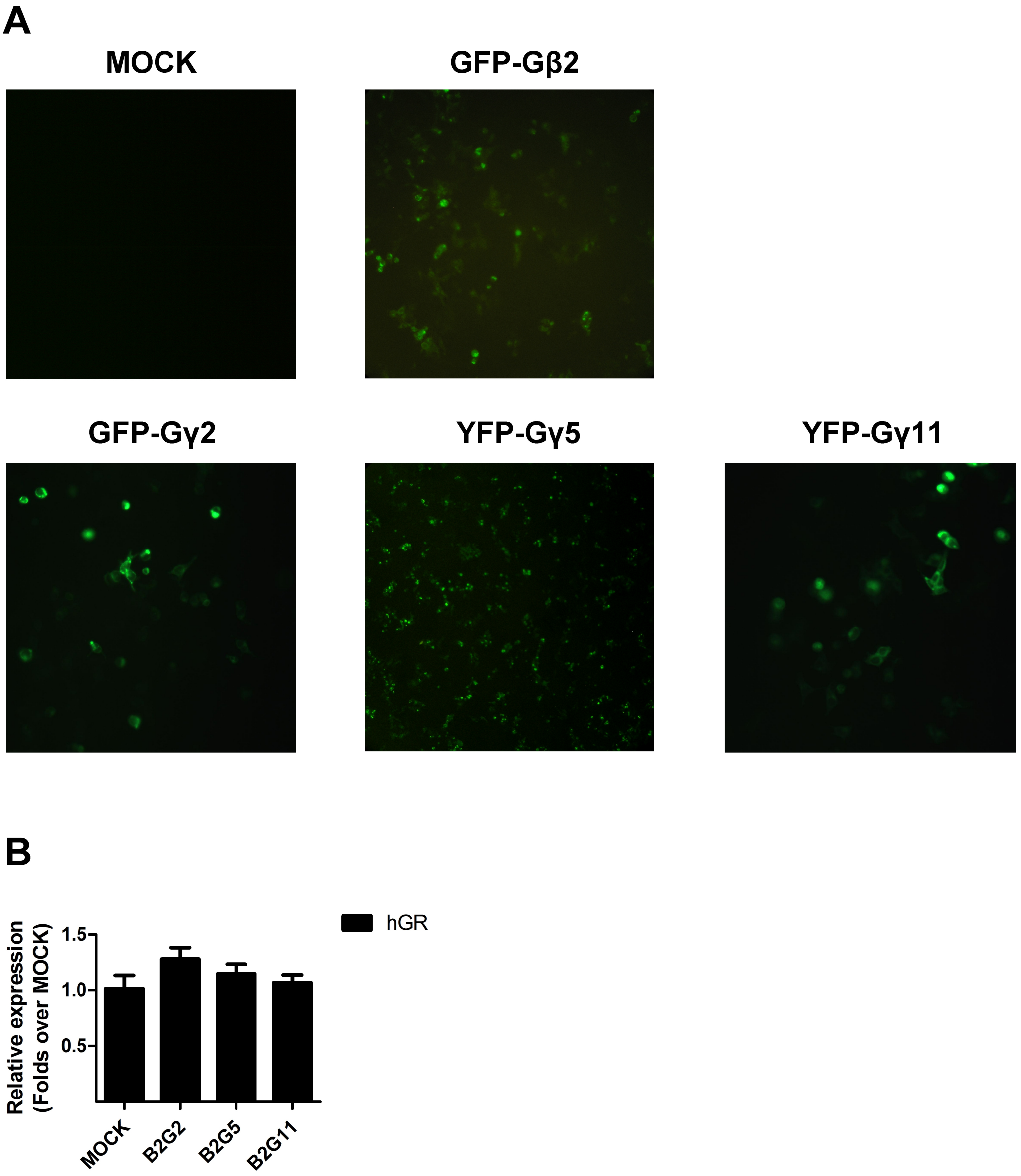

Supplementary figure 4. (A) Representative images of HEK293T cells transfected with GFP-Gβ2, GFP-Gγ2, YFP-Gγ5, and YFP-Gγ11, showing the expression of fluorescent proteins as revealed by fluorescence microscopy. (B). Glucocorticoid receptor mRNA levels measured by quantitaive PCR.

Histamine increases pJNK levels in a Gβγ dependent manner.

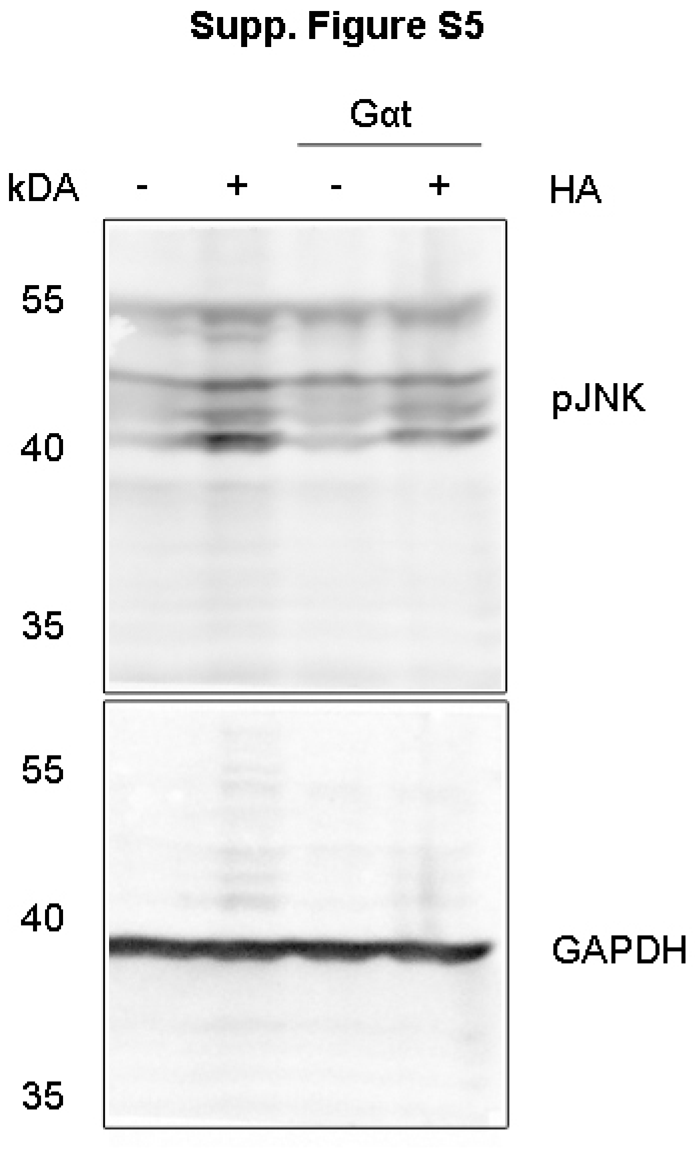

Supplementary figure 5: Full-length blots corresponding to Figure 5.

Histamine potentiates dexamethasone-induced GR activity on MMTV-Luc gene-reporter assay on HEK293T and HeLa cells.

Supp. Figure S6

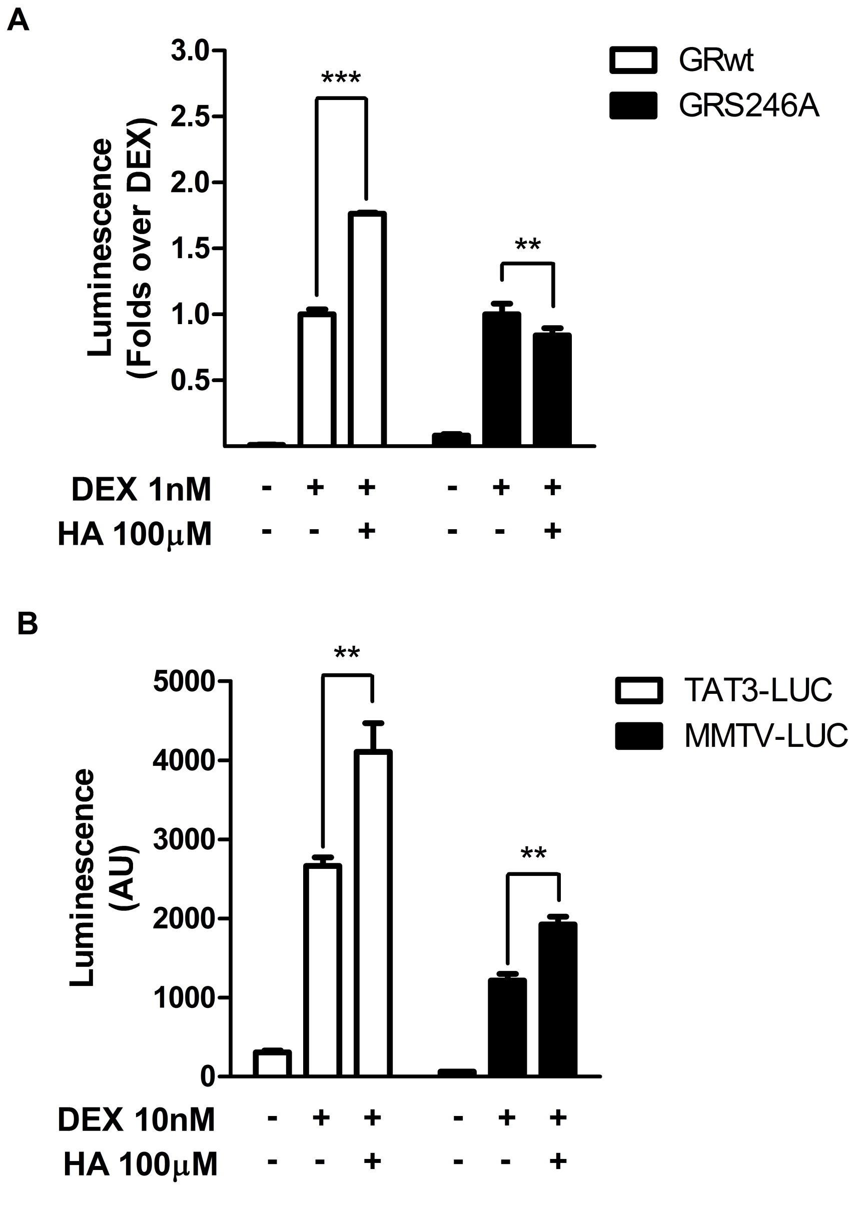

Supplementary figure 6. (A). HEK-293T cells co-transfected with MMTV-Luc and H1R constructs were co-transfected with GR or GR-S246A as indicated and then subjected to treatments **(B).** HeLa cells co-transfected with GR and H1R constructs were co-transfected with TAT3-Luc or MMTV-Luc as indicated and then subjected to treatments. Luciferase activity was determined as stated in methodology section. Results are mean+/-SEM of at least three independent experiments performed in triplicates. **$p<0.01$, ***$p<0.001$.

H1 receptor signaling in A549 cells.

Intracellular Ca^{2+} measurement

Fura 2-AM was used as a fluorescent indicator. Cells were plated on a 96 well plate 24 h before the experiment. To perform the assay, cells were incubated in a buffered saline solution (BSS; 140 mM NaCl, 3.9 mM KCl, 0.7 mM KH 2 PO 4 , 0.5 mM Na 2 HPO 4 Á12H 2 O, 1 mM CaCl 2 , 0.5 mM MgCl 2 , and 20 mM HEPES, 10 mM glucose, and 0.1% BSA, pH 7.5) in the presence of 2 mM Fura 2-AM. Cells were then exposed for 30 min at 37°C in an atmosphere of 5% CO_2, time by which Fura 2-AM was trapped intracellularly by esterase cleavage. Cells were then washed twice in BSS. Fluorescence was measured in a FlexStation3 (MolDev) with the thermostat adjusted to 37°C. Intracellular Ca $^{2+}$ levels were registered every two seconds by exposure to alternating 340-nm and 380-nm light beams, and the intensity of light emission at 505 nm was measured. In this way, light intensities and their ratio (F 340 /F 380) were tracked. Ligands were pipetted in each well without interrupting recording.

Supp. Figure S7

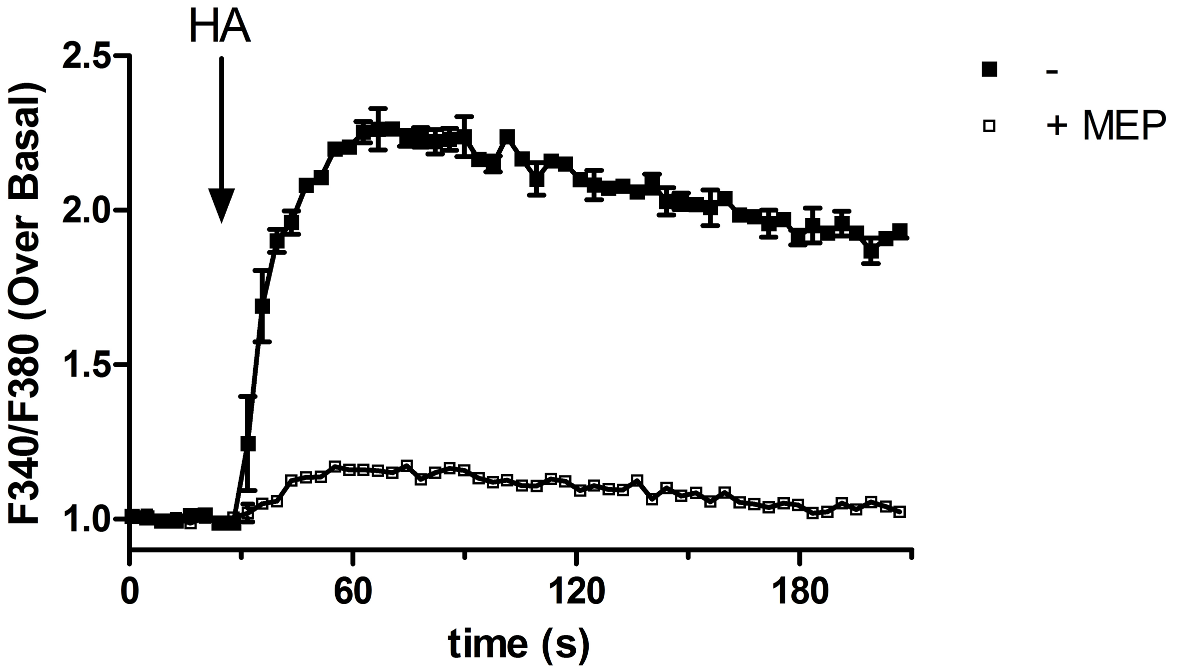

Supplementary figure 7. A549 cells were incubated with Fura-2AM and stimulated with 100µM histamine (HA) when indicated by the arrow, in the absence or presence of 10µMmepyramine (Mep).

TNFα induction of COX-2 mRNA levels in U937 cells.

Supp. Figure S8

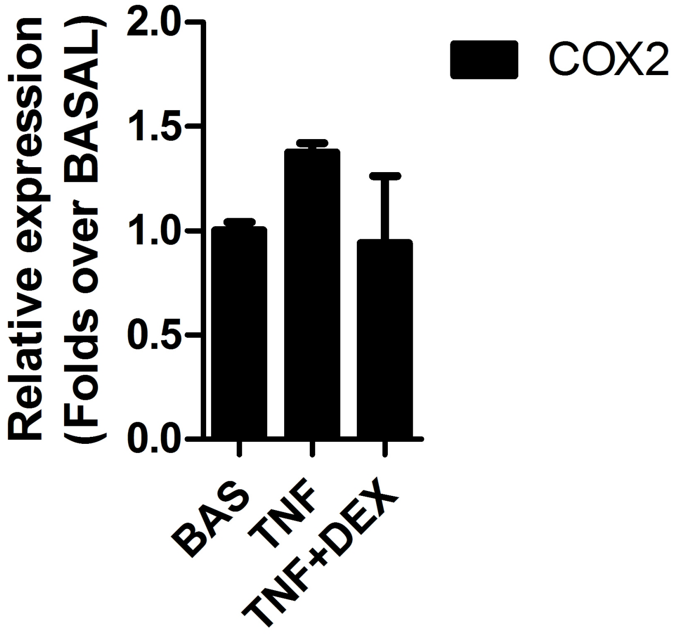

Supplementary figure 8. U937 cells were incubated with TNFα 2000UI/ml for 4 h and with dexamethasone (DEX) for 24 h. COX-2 mRNA levels were quantified by qPCR as indicated in methodology section. No significant differences were found.
